# Techniques of fragile renal organoids transplantation in mice

**DOI:** 10.1590/ACB361102

**Published:** 2021-12-17

**Authors:** Naoto Matsumoto, Kenji Matsui, Yatsumu Saitou, Tsuyoshi Takamura, Shuichiro Yamanaka, Takashi Yokoo, Eiji Kobayashi

**Affiliations:** 1 MD. Division of Nephrology and Hypertension - Department of Internal Medicine - The Jikei University School of Medicine - Tokyo, Japan.; 2 MD. Division of Nephrology and Hypertension - Department of Internal Medicine - The Jikei University School of Medicine - Tokyo, Japan.; 3 MD, PhD. Division of Nephrology and Hypertension - Department of Internal Medicine - The Jikei University School of Medicine - Tokyo, Japan.; 4 MD, PhD. Division of Nephrology and Hypertension - Department of Internal Medicine - The Jikei University School of Medicine - Tokyo, Japan.; 5 MD, PhD. Department of Kidney Regenerative Medicine - The Jikei University School of Medicine - Tokyo, Japan.

**Keywords:** Kidney Transplantation, Cell Aggregation, Organoids, Spinal Cord, Mice

## Abstract

**Purpose:**

This study aimed to develop a microsurgical technique to transplant extremely fragile renal organoids *in vivo*, created by *in-vitro* reaggregation of metanephros from fetal mice. These organoids in reaggregation and development were examined histologically after transplantation under the renal capsule.

**Methods:**

Initially, metanephros from fetal mice were enzymatically treated to form single cells, and spheroids were generated *in vitro*. Under a microscope, the renal capsule was detached to avoid bleeding, and the outer cylinder of the indwelling needle was inserted to detach the renal parenchyma from the renal capsule using water pressure. The reaggregated spheroid was aspirated from the culture plate using a syringe with an indwelling needle outer cylinder and carefully extruded under the capsule. Pathological analysis was performed to evaluate changes in reaggregated spheroids over time and the effects of co-culture of spinal cord and subcapsular implantation on maturation.

**Results:**

*In vitro*, the formation of luminal structures became clearer on day 5. These fragile organoids were successfully implanted without tissue crapes under the renal capsule and formed glomerular. The effect of spinal cord co-transplant was not obvious histrionically.

**Conclusions:**

A simple and easy method to transplant fragile spheroids and renal under the renal capsule without damage was developed.

## Introduction

In the 1950s, small cell aggregates could be generated from animal fetal cells using pellet culture and other methods to autonomously form structures similar to adult tissues *in vitro* 1. This classic finding of self-assembly in cell aggregation is an essential biological phenomenon in the field of regenerative medicine research and an extremely important principle for the creation of organoids and even organs from human stem cells.

Recently, researchers have attempted to self-organize various microtissues and reported that organoids, which are the buds of tissues such as the brain, eye cup, and kidney, can be produced from small cell aggregates generated from human stem cells^2^. To create kidney organoids, it is essential to create cell assemblies that enable cell-cell interactions on a larger scale in multiple cell groups, such as nephron progenitors, ureteric buds, stromal progenitors, and even endothelial progenitors^3^^,^ 4. The contraction of mesenchymal cells may provide an important principle for this cell aggregate to become an organoid, which is the bud of the organ^5^. Furthermore, the original development of the kidney in the same location is noteworthy.

This study established a method to effectively transplant organoids, which are fragile cellular reaggregates, under the renal capsule, in which the kidney originally develops. As previously reported^6^^,^ 7, fetal kidneys were dispersed to a single-cell level and reaggregated *in vitro*, and the degree of development of renal organoids was pathologically verified. The effect of spinal cord co-culture^8^ was also examined. Then, using a modified renal organoid transplantation method, we conducted an *in-vivo* transplantation study to induce further differentiation and maturation under the renal capsule, which has the same location as the original kidney.

## Methods

Fetuses of pregnant B6 mice (purchased from SLC Japan) were used as donors, and mature male NOD/Shi-scid and IL-2RγKOJic mice (NOG mice) (purchased from CLEA Japan) were used as recipients. All animal experiments were approved by the Animal Experiment Committee of Jikei University School of Medicine (permit no. 2021-017). Experiments were conducted in conformance with the National Institutes of Health Guide for the Care and Use of Laboratory Animals. Every effort was made to minimize animal suffering.

### Fetal kidney single cell

Pregnant female B6 mice were used for each spheroid production experiment (N=10). The procedure was performed based on the method by Unbekant *et al*.^6^. Briefly, the steps were as it follows: pregnant B6 (E13) mice were induced with isoflurane inhalation anesthesia, fetuses were harvested, and pregnant mice were euthanized by injection of pentobarbital (120 mg/kg). Removed fetuses were immediately euthanized by decapitation. Fetal mouse metanephroi were harvested from decapitated fetuses under an operating microscope and collected in 1.5-mL tubes containing minimum essential medium α (MEMα) (Cat# 12561056; Gibco). Then, the tubes were centrifuged at 700 G for 3 min, the supernatant was removed, and 1 mL of Accutase at room temperature was dispensed and mixed by vortexing. Next, the cells were incubated for 5 min, pipetted, and agitated again, and centrifuged at 300 G for 5 min. So, the supernatant Accutase was removed. Moreover, 1,000 μL of medium containing 10 μM Y2763 (Cat# 257-00511; Wako) in MEMα with 10% fetal bovine serum (FBS) was added, and the cell component solution was filtered through a 40-μm cell strainer (BD Falcon, Oxford, United Kingdom) to confirm that a single-cell suspension was obtained.

The cell count of this single-cell suspension was determined and adjusted with medium to 1.5 × 106 cells/mL. The cells were divided into 96-well U-bottoms, each containing an average of 3 × 105 cells/well. Finally, the cells were centrifuged at 1,000 rpm for 4 min and incubated under an incubator at 37°C.

### In-vitro culture of renal organoids

**Figure 1:**
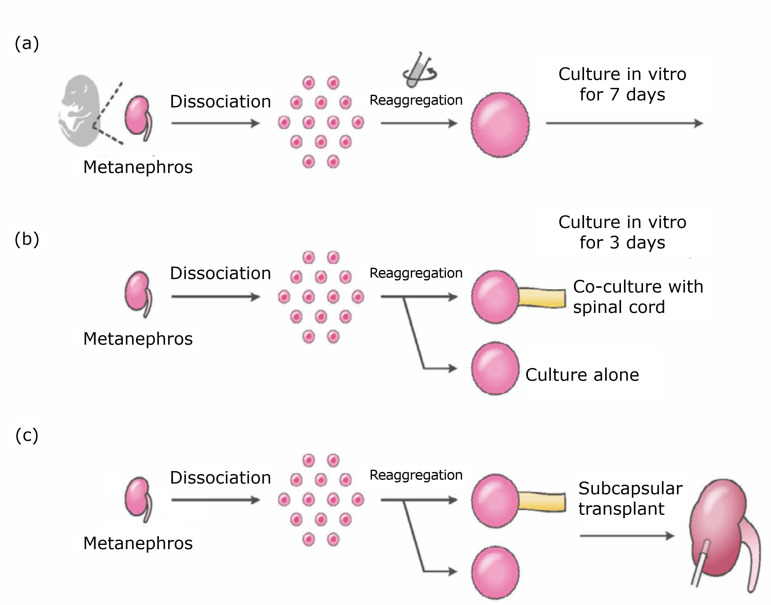
Flowchart of the experimental outline. **(a)** *In-vitro* assay for cell aggregation of fetal renal cells. Fetal metanephros were dispersed by enzymatic treatment. After centrifuging, cell aggregation was cultured for seven days. **(b)** *In-vitro* assay for renal cell aggregation with or without co-culture of spinal cord. Reaggregated spheroids prepared as described before were co-cultured with or without spinal cord for three days to test them for histological maturation. **(c)** *In-vivo* transplantation of reaggregated spheroids under the renal capsule of immunodeficient mice. The reaggregated spheroids prepared as already described were transplanted using the new transplantation method developed in this study.

On the next day (day 1), it was confirmed that the single cells had formed aggregated spheroid. Then, Y27632 was removed from the culture medium, and the cells were cultured in medium supplemented with MEMα + 10% FBS and 1% penicillin/streptomycin for the next six days (until day 7). As the renal organoids form fetal mice were gradually deteriorated in our culture condition after day 7, their observation and sampling were completed at day 7 (Fig. 1a).

Aggregated spheroids were observed under an inverted microscope (Leica DMi1) and obtained for pathological verification on days 1, 3, 5, and 7. Samples were fixed in 4% paraformaldehyde, dehydrated in 20% sucrose, and compounded with optimal cutting temperature (OCT) compound, and sections were prepared at the thickness of 8 μm. Hematoxylin and eosin (HE) staining was performed using standard procedures for histological analysis.

Furthermore, in the separated samples, *in-vitro* experiments were performed to test whether early organoids (Day 1) after cell reaggregation had any effect on differentiation by co-culturing the spinal cord of inbred mice. Fetal mouse spinal cords were harvested from pregnant B6 mice (E12.5) and incubated on polycarbonate filters at both ends of the spheroids aggregated on the air-liquid interface (0.8 mm; Corning Inc., Corning, NY, United States of America) for three days (N=4) (Fig. 1b).

Whole-mount staining was performed to evaluate the number of cap mesenchymes and morphology of the spheroids imaged in the z-stack, both with and without spinal cord co-culture.

#### Transplantation method of newly developed renal organoids

Eight-week-old male immunodeficient NOD/SCID mice (Jackson Immuno Research Laboratories, West Grove, PA, United States of America) were used as recipients for kidney organoid transplantation (N=4). Transplantation of renal capsular was conducted on both left and right kidneys (Fig. 1c).

Organoids are extremely fragile, and various attempts have been made to transplant them into the renal capsule^9^^-^ 12. However, the differences between our new orthotopic transplantation method and other methods are shown in Table 1.

**Table 1:** Previous sub-renal capsular transplantation

Ref. No.	Method	Transpl.
9	An incision was made in the flank, the kidney was exposed outside the body, and a small incision was made in the renal capsule. Kidney organoids were transplanted under the renal capsule.	Kidney organoids
10	The host kidney capsule was incised approximately 2 mm from the caudal end of the kidney, and the rod was carefully inserted laterally with forceps. The inserted rod was positioned to create a V-shaped free space, and the capsule membrane was briefly cauterized with electrocautery to prevent deviation from the initial position. Finally, iPS-derived aggregates containing the spinal cord of mouse embryos and mixture spheres were inserted through the incised window using a 20-gaugeplastic indwelling needle connected to a P-200 Gilson pipette.	HUVEC+MSC Agarose gel rod iPS-derived aggregates
11	The kidneys were exposed through an incision on the dorsal flank. After making an incision of approximately 2 mm in the host kidney capsule with a 23-gauge needle, a PE50 tube containing 10-20 kidney organoids was carefully placed under the kidney capsule. The kidney organoids were injected by carefully blowing from the other side of the PE50 tube.	Kidney organoids
12	Left kidney was exteriorized, and a small insertion was made in the renal capsule. Kidney organoids were transplanted onto the left kidney capsule of mice using 24 Ghz catheter.	Kidney organoids

iPS: Induced pluripotent stem cells; HUVEC: human umbilical vein endothelial cells; MSC: mesenchymal stem cells. Tranpl.: Transplantation

#### Transplantation position

First, the recipient mouse was anesthetized with isoflurane inhalation, and a 1.5-cm midline abdominal incision was made.

Although there have been reports of transplantation without laparotomy using a posterior-dorsal approach, in this method, the recipient was transplanted under the microscope through laparotomy.

#### Renal capsule pocket creation method

**Figure 2:**
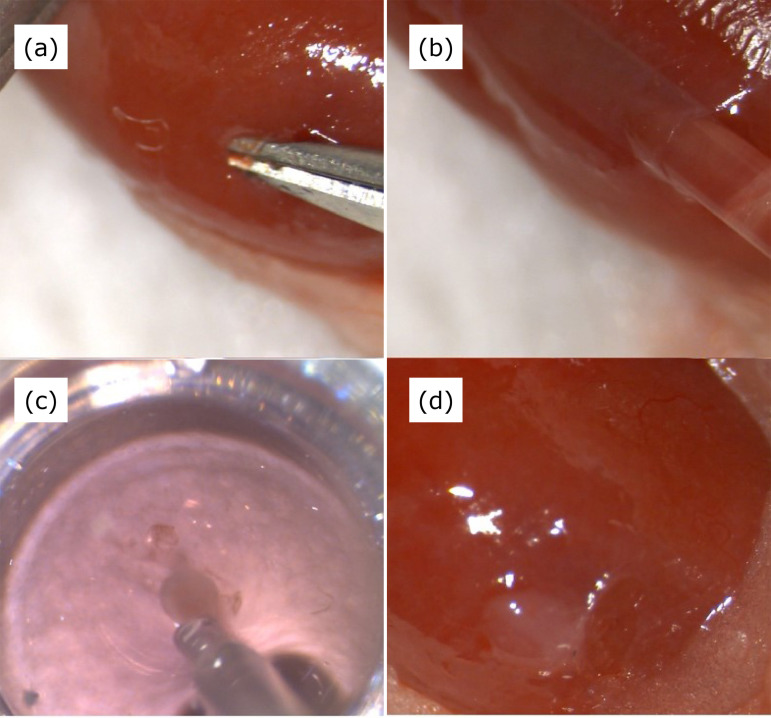
Newly developed method of spheroid implantation into the renal capsule. **(a)** Incision and dissection of the renal capsule. **(b)** Insertion of a perfusion tube under the renal capsule. **(c)** Inhalation of reaggregated spheroid. **(d)** Post-implantation of spheroid.

The intestine was moved to the left or right to expose the kidney, and the renal capsule in the lower part of the kidney was detached by approximately 1 mm using the micro-shear (Fig. 2a). The tip of a 20-G Surflo needle was cut at an angle. The Surflo needle with a smooth cutting surface to prevent damage to the renal detachment surface was gently inserted through the incision in the capsule, and detachment was performed while adding a small amount of saline and keeping the cutting surface facing the renal parenchyma (Fig. 2b).

The water pressure of the syringe was adjusted by pressing the syringe with a hand. In case of poor detachment, the Surflo needle should not be pushed in, but a cotton swab should be pushed in while guiding the coated side. This manipulation does not cause even the smallest amount of bleeding and does not leave any blood in the peeling pocket. Then, the spheres were inhaled in culture in a 96-well U-bottom using the same 20-G Surflo needle outer tube (Fig. 2c). For aspiration, culture medium was aspirated as little as possible as if turning a push syringe, and the organoid was positioned at the leading edge of the Surflo tube. Again, the same Surflo needle was positioned into the incision, and the fragile renal organoid was gently implanted into the pocket (Fig. 2d). Then, the incision was closed with a 5-0 thread running suture.

Postoperatively, recipient mice were sacrificed to death on day 7 of transplantation, and pathology was harvested after tissue confirmation under magnification of the transplantation site.

### Whole-mount staining

Cultured spheres were fixed in 4% paraformaldehyde for 60 min at 4°C and washed three times with PBS. Samples were blocked with 1% donkey serum, 0.2% skim milk, and 0.3% Triton X/PBS for 1 h at room temperature and incubated with primary antibodies overnight at 4°C. After washing three times with PBS, samples were incubated with secondary antibodies conjugated with Alexa Fluor 488, 546, and 647 for 1 h at room temperature. The samples were incubated for 1 h at room temperature. The samples were mounted with ProLong Gold mounting medium containing 4′,6-diamidino-2-phenylindole (DAPI). Each sample was observed under a fluorescence microscope (LSM 880 Confocal; Carl Zeiss, Munich, Germany).

## Results

### In-vitro reaggregation sphere formation of fetal kidney cells

**Figure 3:**
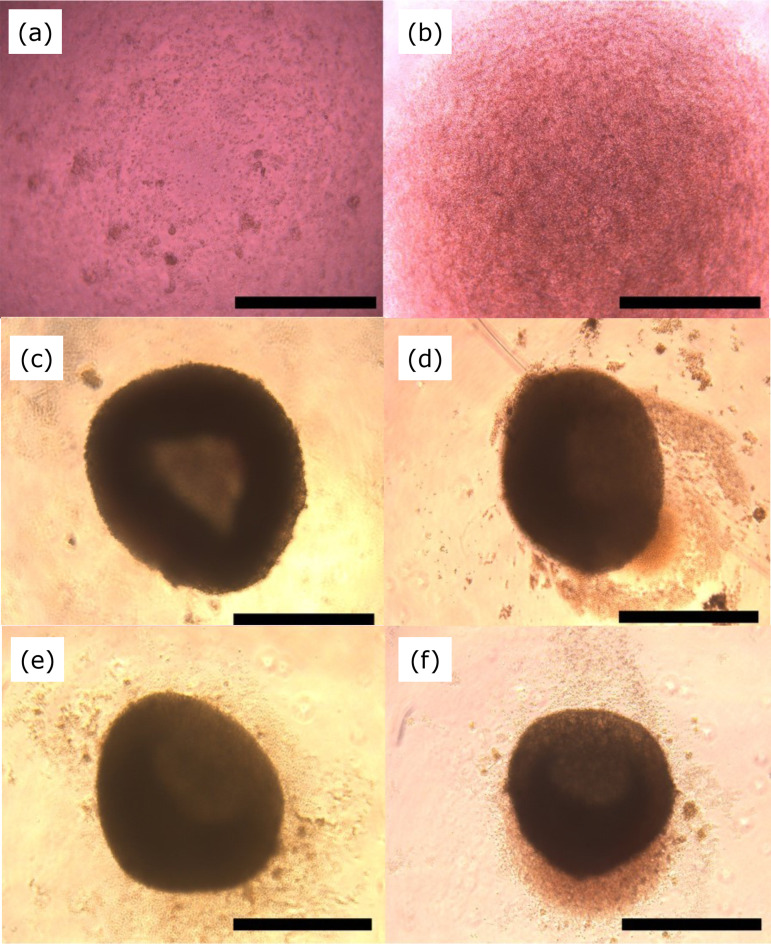
Changes in reaggregated spheroid from fetal kidney over time. Bright field photograph of the 96-well culture. **(a)** Day 0 (before centrifugation). **(b)** Day 0 (after centrifugation). **(c)** Day 1. **(d)** Day 3. **(e)** Day 5. **(f)** Day 7. Scale: 1 mm.

*In-vitro* reaggregation of single cells was performed by centrifugation (Fig. 3 a,b). Moreover, 1-mm diameter spheroids were formed on day 1 (Fig. 3c). After seven days of incubation, no increase in the size of the cell aggregates was observed (Fig. 3 d-f).

HE staining was performed on days 1, 3, 5, and 7, and no significant structures were formed in the center, but tubular structures and surrounding cells formed cap mesenchymes at the outer edge.

**Figure 4:**
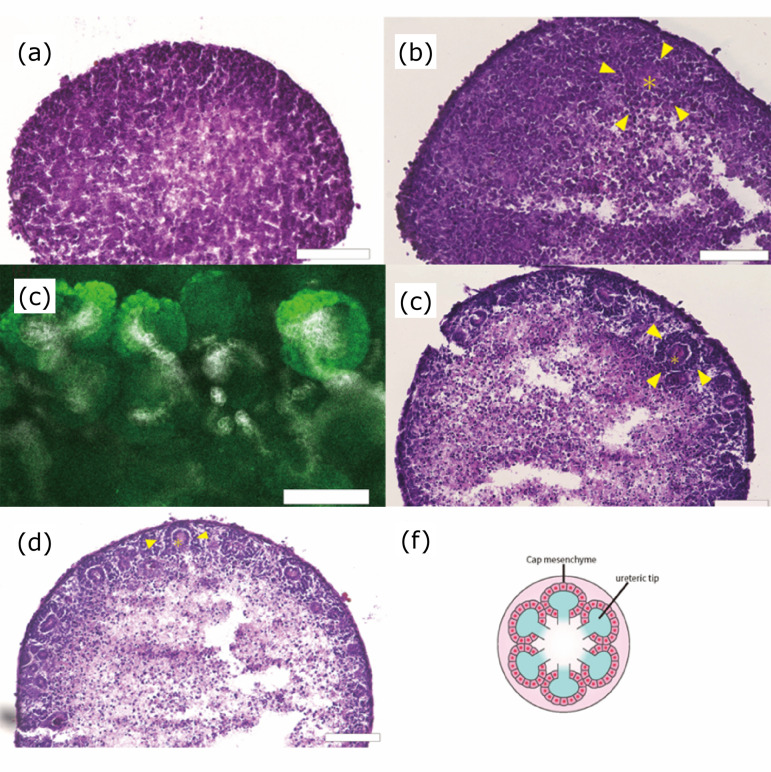
Time course of reaggregated spheroid from fetal kidney. HE stained photograph and immunostaining. **(a)** Day 1. **(b)** Day 3. **(c)** Day 5. **(d)** Day 7. HE staining. Scale bar: 100 μm. **(e)** Day 7 (immunostaining). Scale bar: 100 μm. Six2 (green), CK8 (white). **(f)** Organogram of reaggregated renal spheroids. On day 1, the cells were agglutinated, but no significant structures could be morphologically identified. On day 3, immature structure of cap mesenchyme and ureteric tips was detected, following day 5 and day 7, these structures could be clearly observed.

HE: hematoxylin and eosin; *ureteric buds; arrow: cap mesenchyme Six2 (green) and CK8 (white) in immunostaining.

As shown in Fig. 3c, the cell agglutination was observed on day 1, but histological examination showed no significant cell structure formation by use of HE staining (Fig. 4a). While fetal metanephros have been known that cap mesenchymes might be formed around the ureteric bud, the reaggregated spheroid obtained from day 3 showed that the formation of ureteric buds (*) and cap mesenchymes (arrows) could start to be confirmed (Fig. 4b). Those morphological findings were clearer in the samples obtained from day 5 and day 7 (Fig. 4 c,d).

At day 7, the distribution of nephron progenitor cells and ureteric buds was evaluated by whole immunostaining for Six2 and CK8. Multiple cap mesenchyme formation was observed around the ureteric buds. The ureteric buds with surrounding cap mesenchyme structures were spherical and branched, but the ureteric buds present alone had only spherical structures (Fig. 4e).

**Figure 5:**
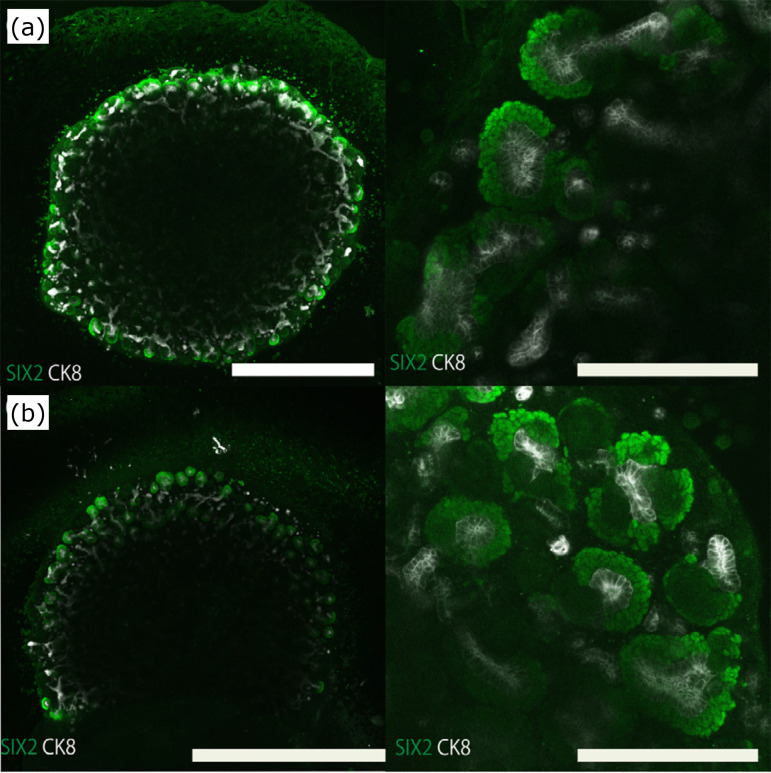
Effect of spinal cord on mouse metanephros reaggregation spheroid *in vitro*. **(a)** Whole immunostained image of mouse reaggregated spheroid and spinal cord after three days of co-culture (left, low magnification; right, high magnification). **(b)** Whole immunostained image of mouse reaggregated spheroid after three days in culture alone (left, low magnification; right high magnification). Scale bar (low magnification: 1 mm, high magnification: 200 μm).

The effect of *in-vitro* co-culture of kidney spheroids and spinal cord co-culture of kidney spheroids with the spinal cord is known to trigger epithelialization of metanephric mesenchyme and formation of luminal structures^8^. However, to evaluate the effect on reaggregation, immunostaining was performed after three days of culture. As a result, there was no significant difference in the number of cap mesenchymes and ureteric buds between spinal cord co-culture and sphere alone (Fig. 5), neither in their morphology.

In-vivo *development of reaggregates in the new transplantation method*

Soft reaggregates on day 2 of reaggregation were transplanted into the renal capsule using a new technique that reduced damage to the capsule.

**Figure 6:**
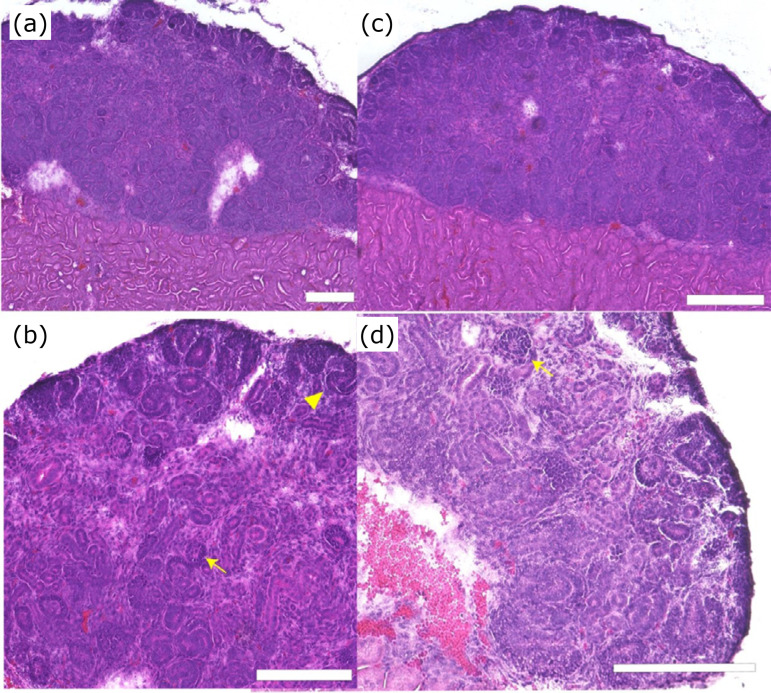
Effect of spinal cord on mouse metanephros reaggregation spheroid during transplantation. **(a)** HE stained image of mouse metanephros reaggregation spheroid transplanted alone (low magnification). **(b)** High magnification. **(c)** HE stained images of mouse spinal cord and mouse metanephros reaggregation spheroid transplanted alone low magnification. **(d)** High magnification. Scale bar: 200 μm. In the center of the tissue, glomeruli and multiple tubular structures can be observed. There were no significant differences between spheroids implanted with or without the spinal cord.

HE: hematoxylin and eosin; arrowhead: glomerulus; arrow: S-shaped body cap mesenchyme and S-shaped structures can be detected on the periphery of the spheroid.

Even when the aggregated spheroids were cultured *in vitro* for seven days, cap mesenchyme structure alone could be observed morphologically. However, after transplantation of them under the renal capsule, mature structures such as multiple glomerular structures (arrowhead) and S-shaped structures (arrow) were detected (Fig. 6 a,b).

Moreover, erythrocyte infiltration was observed in the stromal region, suggesting that blood vessels had invaded into the graft from host.

There were no granulation-like tissues, nor evidence of damage associated with the renal subcutaneous graft transplantation.

Similar structures were also observed when the spinal cord was transplanted under the renal capsule with spheroids. Glomeruli were observed in the area indicated by arrows. Many other luminal structures can be seen (Fig. 6 c,d). No histological differences were observed in spinal cord co-culture.

## Discussion

In this study, we initially examined reaggregation and differentiation maturation of single-cell fetal kidneys *in vitro*. As previously reported, single-cell fetal kidneys reaggregated to form spheroids after one day of *in-vitro* culture. These spheroids showed clearer formation of luminal structures from day 5, with many reconstituted uretic buds and some cap mesenchyme formation around the ureteric buds.

During nephrogenesis, metanephric mesenchyme is epithelialized and forms a lumenal structure by receiving growth factor signals from ureteric buds. Wnt-4 is involved in one of these signals, and Kispert *et al*.^8^ reported that secretion of Wnt-4 or a similar factor from the dorsal side of the spinal cord causes differentiation of nephron progenitor cells into tubules. *In-vivo* transplantation of organoids obtained by co-culture of nephron progenitor cells and spinal cord also tends to induce the formation of mature glomeruli^10^^,^ 13. However, the co-culture of reaggregated spheres from single-cell mouse fetal kidneys and spinal cord used in this experiment did not show obvious differences in the number and morphology of cap mesenchymes. This is because both ureteric buds and nephron progenitor cells were present in the reaggregating spheres and interacted with each other by receiving signals from each other.

Next, we examined the site of *in-vivo* cell aggregate implantation. However, unlike tissue transplantation, the reaggregates are extremely fragile, and it was necessary to devise a transplantation method. In previous studies, we have reported the convenience of orthotopic transplantation^14^^,^ 15. The method we developed in the previous study was also selected for orthotopic transplantation under the renal capsule, but we established a method to detach the capsule, create a pocket space, and transplant the fragile organoids under water pressure control. This method of transplanting the fragile organoids into the recipient with water flow has been applied into a laparoscopic transplantation device (unpublished).

## Conclusions

In this study, we applied the same principles to develop a technique to transplant soft renal organoids under the renal capsule without damage. When transplanting under the renal capsule, bleeding can easily occur because the kidney is an organ rich in blood vessels. Therefore, in the transplantation method developed this time, the renal capsule and renal parenchyma were detached beforehand by water pressure to avoid hemorrhage and create a space between the capsule and parenchyma, allowing the spheroid to grow without pressure drainage. As shown in our previous study, reaggregated spheroids formed glomeruli by subepithelial transplantation, which was not observed *in vitro*, and it was confirmed that subepithelial transplantation increased the degree of maturation.

We have used metanephros as a scaffold to develop chimeric kidneys by subepithelial transplantation of allogeneic and heterologous nephron progenitors^16^^-^ 18. In the future, we will use the reaggregating spheroid transplantation method developed in this study to verify the appropriate cell ratio in chimeras and the factors that strongly generate interactions.

## References

[1] Moscona A, Moscona H. (1952). The dissociation and aggregation of cells from organ rudiments of
the early chick embryo. J Anat.

[2] Sasai Y (2013). Cytosystems dynamics in self-organization of tissue
architecture. Nature.

[3] Takebe T, Enomura M, Yoshizawa E, Kimura M, Koike H, Ueno Y, Matsuzaki T, Yamazaki T, Toyohara T, Osafune K, Nakauchi H, Yoshikawa HY, Taniguchi H. (2015). Vascularized and complex organ buds from diverse tissues via
mesenchymal cell-driven condensation. Cell Stem Cell.

[4] Takasato M, Er PX, Chiu HS, Maier B, Baillie GJ, Ferguson C, Parton RG, Wolvetang EJ, Roost MS, Chuva de, Little MH (2015). Kidney organoids from human iPS cells contain multiple lineages
and model human nephrogenesis. Nature.

[5] Takebe T, Sekine K, Enomura M, Koike H, Kimura M, Ogaeri T, Zhang RR, Ueno Y, Zheng YW, Koike N, Aoyama S, Adachi Y, Taniguchi H. (2013). Vascularized and functional human liver from an iPSC-derived
organ bud transplant. Nature.

[6] Unbekandt M, Davies JA (2010). Dissociation of embryonic kidneys followed by reaggregation
allows the formation of renal tissues. Kidney Int.

[7] Leclerc K, Costantini F. (2016). Mosaic analysis of cell rearrangements during ureteric bud
branching in dissociated/reaggregated kidney cultures and in
vivo. Dev Dyn.

[8] Kispert A, Vainio S, McMahon AP (1998). Wnt-4 is a mesenchymal signal for epithelial transformation of
metanephric mesenchyme in the developing kidney. Development.

[9] van den Berg CW, Ritsma L, Avramut MC, Wiersma LE, van den Berg BM, Leuning DG, Lievers E, Koning M, Vanslambrouck JM, Koster AJ, Howden SE, Takasato M, Little MH, Rabelink TJ (2018). Renal subcapsular transplantation of PSC-derived kidney organoids
induces neo-vasculogenesis and significant glomerular and tubular maturation
in vivo. Stem Cell Reports.

[10] Sharmin S, Taguchi A, Kaku Y, Yoshimura Y, Ohmori T, Sakuma T, Mukoyama M, Yamamoto T, Kurihara H, Nishinakamura R (2016). Human induced pluripotent stem cell-derived podocytes mature into
vascularized glomeruli upon experimental transplantation. J Am Soc Nephrol.

[11] Subramanian A, Sidhom EH, Emani M, Vernon K, Sahakian N, Zhou Y, Kost-Alimova M, Slyper M, Waldman J, Dionne D, Nguyen LT, Weins A, Marshall JL, Rosenblatt-Rosen O, Regev A, Greka A (2019). Single cell census of human kidney organoids shows
reproducibility and diminished off-target cells after
transplantation. Nat Commun.

[12] Nam SA, Seo E, Kim JW, Kim HW, Kim HL, Kim K, Kim TM, Ju JH, Gomez IG, Uchimura K, Humphreys BD, Yang CW, Lee JY, Kim J, Cho DW, Freedman BS, Kim YK. (2019). Graft immaturity and safety concerns in transplanted human kidney
rganoids. Exp Mol Med.

[13] Tajiri S, Yamanaka S, Fujimoto T, Matsumoto K, Taguchi A, Nishinakamura R, Okano HJ, Yokoo T (2018). Regenerative potential of induced pluripotent stem cells derived
from patients undergoing haemodialysis in kidney
regeneration. Sci Rep.

[14] Kinoshita Y, Iwami D, Fujimura T, Kume H, Yokoo T, Kobayashi E. (2021). Techniques of orthotopic renal transplantation in pigs. One donor
to two recipients via inverted grafting. Acta Cir Bras.

[15] Takamura T, Sasaki H, Hirayama H, Kiyoshi A, Inoue M, Matsui K, Matsumoto N, Saito Y, Fujimoto T, Tajiri S, Yamanaka S, Matsumoto K, Miyawaki T, Yokoo T, Kobayashi E. (2021). Techniques of orthotopic renal transplantation. II. Size-matched
porcine grafts in monkey recipients. Acta Cir Bras.

[16] Yamanaka S, Tajiri S, Fujimoto T, Matsumoto K, Fukunaga S, Kim BS, Okano HJ, Yokoo T. (2017). Generation of interspecies limited chimeric nephrons using a
conditional nephron progenitor cell replacement system. Nat Commun.

[17] Fujimoto T, Yamanaka S, Tajiri S, Takamura T, Saito Y, Matsumoto K, Takase K, Fukunaga S, Okano HJ, Yokoo T. (2019). In vivo regeneration of interspecies chimeric kidneys using a
nephron progenitor cell replacement system. Sci Rep.

[18] Fujimoto T, Yamanaka S, Tajiri S, Takamura T, Saito Y, Matsumoto N, Matsumoto K, Tachibana T, Okano HJ, Yokoo T. (2020). Generation of human renal vesicles in mouse organ niche using
nephron progenitor cell replacement system. Cell Rep.

